# A local outbreak of dengue caused by an imported case in Dongguan China

**DOI:** 10.1186/1471-2458-12-83

**Published:** 2012-01-26

**Authors:** Hong-Juan Peng, Hui-Bing Lai, Qiao-Li Zhang, Ba-Yi Xu, Hao Zhang, Wen-Hua Liu, Wei Zhao, Yuan-Ping Zhou, Xin-Guang Zhong, Shu Jiang, Jin-Hua Duan, Gui-Yun Yan, Jian-Feng He, Xiao-Guang Chen

**Affiliations:** 1 Key Laboratory of Prevention and Control for Emerging Infectious Diseases of Guangdong Higher Institutes, School of Public Health and Tropical Medicine, Southern Medical University, Guangzhou, Guangdong 510515, Peoples Republic of China; 2 Guangdong Centre for Disease Control and Prevention Field Epidemiology Training Program of Guangdong Province, Guangzhou, Guangdong 510300, Peoples Republic of China; 3 Dongguan Centre for Disease Control and Prevention, Dongguan, Guangdong 523129, Peoples Republic of China; 4 Department of Infectious Diseases, Nanfang Hospital, Southern Medical University, Guangzhou, Guangdong 510515, Peoples Republic of China; 5 Program of Public Health, School of Medicine, University of California, Irvine, CA 92697, USA

**Keywords:** Dengue, Epidemiology outbreak, Urbanization

## Abstract

**Background:**

Dengue, a mosquito-borne febrile viral disease, is found in tropical and sub-tropical regions around the world. Since the first occurrence of dengue was confirmed in Guangdong, China in 1978, dengue outbreaks have been reported sequentially in different provinces in South China transmitted by^.^peridomestic *Ae. albopictus *mosquitoes, diplaying *Ae. aegypti*, a fully domestic vector that transmits dengue worldwide. Rapid and uncontrolled urbanization is a characteristic change in developing countries, which impacts greatly on vector habitat, human lifestyle and transmission dynamics on dengue epidemics. In September 2010, an outbreak of dengue was detected in Dongguan, a city in Guangdong province characterized by its fast urbanization. An investigation was initiated to identify the cause, to describe the epidemical characteristics of the outbreak, and to implement control measures to stop the outbreak. This is the first report of dengue outbreak in Dongguan, even though dengue cases were documented before in this city.

**Methods:**

Epidemiological data were obtained from local Center of Disease Control and prevention (CDC). Laboratory tests such as real-time Reverse Transcription Polymerase Chain Reaction (RT-PCR), the virus cDNA sequencing, and Enzyme-Linked immunosorbent assay (ELISA) were employed to identify the virus infection and molecular phylogenetic analysis was performed with MEGA5. The febrile cases were reported every day by the fever surveillance system. Vector control measures including insecticidal fogging and elimination of habitats of *Ae. albopictus *were used to control the dengue outbreak.

**Results:**

The epidemiological studies results showed that this dengue outbreak was initiated by an imported case from Southeast Asia. The outbreak was characterized by 31 cases reported with an attack rate of 50.63 out of a population of 100,000. *Ae. albopictus *was the only vector species responsible for the outbreak. The virus cDNA sequencing analysis showed that the virus responsible for the outbreak was Dengue Virus serotype-1 (DENV-1).

**Conclusions:**

Several characterized points of urbanization contributed to this outbreak of dengue in Dongguan: the residents are highly concentrated; the residents' life habits helped to form the habitats of *Ae. albopictus *and contributed to the high Breteau Index; the self-constructed houses lacks of mosquito prevention facilities. This report has reaffirmed the importance of a surveillance system for infectious diseases control and aroused the awareness of an imported case causing the epidemic of an infectious disease in urbanized region.

## Background

Dengue, a mosquito-borne febrile viral disease, as per the World Health Organization (WHO) estimates, is now prevalent in over 100 countries in Africa, the Americas, the Eastern Mediterranean, Southeast Asia and the Western Pacific, posing a threat to more than 1.8 billion people in the tropics and subtropics [1,2], and the infected population is about 100 million every year [2]. The viruses of dengue belong to the family Flaviridae and include four serotypes (DENV-1, -2, -3, -4), all of which can classically cause undifferentiated fever [2,3].

In China, the first outbreak of dengue was confirmed in Guangdong province in 1978. Since then, dengue epidemics were reported sequentially in Hainan, Guangxi, Fujian, Zhejiang and Yunnan provinces [1]. *Ae. albopictus *a peridomestic mosquito that is known to be the only vector of dengue in the Pearl River Delta area in Guangdong province [4,5], displacing *Ae. aegypti*, the fully domestic dengue vector [6]. Guangdong is located in a subtropical zone, with characteristic humidity and hot weather. The summer is long, lasting from May to October, and the average annual rainfall is 1,700 mm. In recent years, Guangdong province has had a high incidence of dengue epidemics, with cases reported every year since 1995 [1,7]. Even though, Donggue is free of dengue outbreak and this is the first report of dengue outbreak in Dongguan. Urbanization is a characteristic change in developing countries, which contributes to dengue endemic factors including vector breeding area development, high resident density and life habits, and virus transmission dynamics [8]. Dongguan is characterized by its fast urbanization in south China. This city is located in the Pearl River Delta area and mid-south of Guangdong province. Dongguan has an area of 2465 km^2^, and a population of 6.35 million including 5.35 million immigrants. In September 2010, an outbreak of dengue transmitted by *Ae. albopictus *happened in Dongguan China. An investigation was thus initiated to trace the source, describe the epidemiological characteristics, and implement control measures to stop the dengue outbreak.

## Methods

### Ethics statement

When evaluated by the Institutional Review Board of the Guangdong province CDC, this non-research activity was considered a component of the public health response to the dengue outbreak in Dongguan and thus did not require review. Data analysis was performed on an anonymous dataset preserved in the Guangdong province CDC electronic reporting system.

### Data sources

As dengue is a notifiable disease in China, hospitals and clinics are required to report clinical diagnosed and laboratory confirmed cases of dengue first to the county CDC, and then to the provincial CDC. On Sep 3, 2010, the first diagnosed dengue case hospitalized in Qifeng clinic in Dongguan on Aug. 23, 2010, was reported to the local CDC. When this notification was received, the province CDC organized and performed laboratory verification, source tracing, and epidemiological investigation such as searching for other dengue cases or clusters.

### Source tracing and public awareness of the outbreak

On Sep 3, 2010, the first case of Dengue was found and confirmed with serum antibody detection. From then on, all of the health care organizations in Dongguan city were informed to be aware of the outbreak and all of the febrile cases were reported to the province CDC. The representatives from the public health system went into the community for site investigation and vector control, especially for teaching of the prevention methods to the residents. The Medias were also involved to the public awareness.

### Case definitions

According to the diagnostic criteria for Dengue Fever (DF) (WS216-2008) enacted by the Ministry of Health of People's Republic of China[9], a suspected case of DF had (1) the experience of being in the dengue endemic area within 14 days before symptoms appearing; (2) at least two of the symptoms as follows: acute onset of fever, severe headache, orbital pain, myalgia, arthralgia, fatigue, flush, rash, conjunctival congestion; If not (1), but (2) and with leucopenia or thrombocytopenia. The blood specimens from the suspected patients were collected during the acute phase (within 7 days after symptom onset) for laboratory diagnosis.

A clinically diagnosed case was the suspected case with leucopenia or thrombocytopenia, and lived in Xintang, Dongguan (where the first confirmed case was found); or the suspected case with IgG or IgM positive in serum detection.

A laboratory confirmed case was the suspected case if dengue virus (DENV) Ribonucleic Acid (RNA) was detected in the serum by real-time PCR; or if virus was isolated from the acute infection patient's blood, tissue or cerebrospinal fluid; or if the IgG titer in recovery period was 4 times higher than that in acute period.

### Laboratory diagnosis and virus identification

All laboratory diagnosis was performed at the Guangdong province CDC and Southern Medical University. A real-time RT-PCR assay was used to identify virus in serum specimens collected within 7 days from the onset of fever (acute-phase serum specimen) [10,11]. The patients' acute serum was inoculated to suckling mice's cranial cavity. The brain homogenate of the sick mouse was then inoculated to *Ae. albopictus *mosquito C6/36 cells to get the amplified virus. The TRIzol^® ^reagent (GIBCOBRL Cat# 15596) was used to extract the total RNA from the supernatant of the culturing DENV infected C6/36 cells. The Prime Script^RT ^reagent kit ^® ^(Takara, Cat # DRR037A) was used in transcription of the DENV RNA, and SYBR^® ^Premix (Takara, Cat # DRR411) was used in real-time PCR, and the information of primers, probes and program is shown in Table 1. The virus RNA was transcribed and sequenced.

**Table 1:** Real-time PCR primers and probe for dengue virus cDNA identification*

Primers/probe	Sequence (5'-3')	Working concentration	Length of amplified fragment (bp)	Type specificity
Den-FP	GCATATTGACGCTGGGAGAGA	0.5 μM	68	Universal for all four types
		
Den-RP	GGCGTTCTGTGCCTGGAAT	0.5 μM		
		
Den-probe	**FAM**CAGAGATCCTGCTGTCTC**MGB**	0.25 μM		
Real-time PCR program	Pre-denature at 95°C for 45 s, denature at 95°C for 20 s, annealing at 65°C for 20 s, elongation at 72°C for 30 s, 40 cycles, elongation at 72°C for 1 min, keep at 4°C			

*From diagnostic criteria for Dengue Fever (DF) (WS216-2008) enacted by the Ministry of Health of People's Republic of China.

The phylogenetic analysis was conducted building a dataset including, the E gene sequence D130DG isolate in Guangdong province, plus 140 E gene reference sequences downloaded after the BLAST analysis from NCBI Database (http://www.ncbi.nlm.gov/BLAST/). The reference sequences were selected on the basis of the following inclusion criteria: 1) the sequences had to be already published in peer-reviewed journals; 2) there had to be no uncertainty about the subtype assignment of each sequence and all were classified as non-recombinant; 3) the city/state of origin and sampling date were known and clearly established in the original publication. All the sequences were aligned with the Clustal X algorithm [12] and then were manually edited using Bioedit v7.0 [13]. Positions containing gaps were removed from the final alignment. Maximum likelihood (ML) phylogenetic trees were inferred with Mega5 program [14] using the GTR + G + I nucleotide substitution model, which was selected with the hierarchical likelihood ratio test described by Swofford and Sullivan[15]. The statistical robustness and reliability of the branching order within each phylogenetic tree were confirmed with a bootstrap analysis using 1000 replicates.

The density of white cells and thrombocytes in the whole blood from the suspected patients was detected. Serum from the suspected patients was detected for IgG and IgM antibodies with ELISA kit (Dengue IgG Indirect ELISA, Cat No. E-DEN01G; Dengue IgM Indirect ELISA, Cat No. E-DEN01M) [7].

### Fever surveillance

A fever is any body temperature elevation over 100°F (37.8°C). The fever surveillance system is composed of all of the clinics, outpatients of the hospitals and affiliated to the Health system in China. It implements the ordinary fever surveillance such as monitoring the fever cases and especially the outbreak of fever cases. When the outbreak of dengue was reported in Xintang community of Dongguan, Qifeng clinic which is the nearest to Xintang community, was chosen to be a special observation site for the febrile cases surveillance. From Sep.3 to Oct. 26, the total number of the febrile cases and the suspected dengue cases was reported to CDC system every day.

### Dengue vector monitoring

Vector monitoring system is affiliated to the health system, and performs a monthly investigation for mosquito density. After the dengue case was reported, the investigation for *Ae. albopictus *habitats and Breteau Index (BI)--number of positive containers for *Aedes *per 100 houses [7] were carried out every day in Xintang community from Sep. 10, 2010 to Sep. 28, 2010. The staffs from CDC system and the health system implemented the site investigation in residents' home and neighborhoods, and instruction of dengue prevention to the residents.

## Results

### Description of outbreak

In this dengue outbreak, 31 cases were diagnosed and reported, 7 of which were clinically diagnosed and 24 were laboratory confirmed. Some of the reported cases exhibited clustering that we found 2 cases in one family, and 3 in another family. The epidemic was restrained in the village of Xintang community in Dongguan city, including 3 residents' groups. These groups are closely distributed with a dense population. This community has an area of 4.33 km^2 ^and a population of 61,233, with 11,233 residents and 50,000 immigrants. The housing is mostly low-rise self-constructed buildings that are regularly distributed and separated by 1-2 m wide alleyways. Houses lack window screen to prevent mosquitoes, and the residents have a habit of cultivating water plants and storing water in their homes for plants watering. The hygiene situation is generally good.

**Figure 1:**
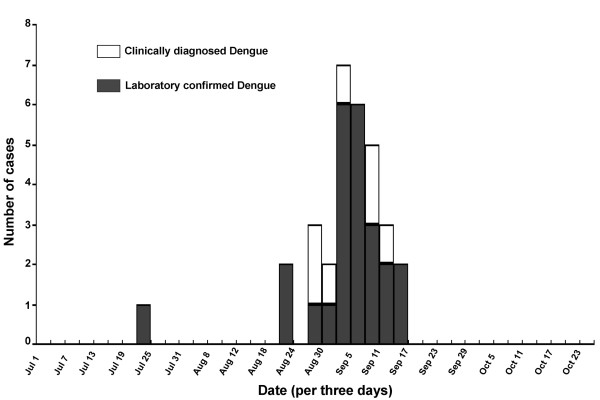
**Number of clinically diagnosed and laboratory confirmed dengue cases at different time points during the dengue outbreak**. The outbreak of dengue occurred from July 22, 2010 to September 14, 2010, with a concentrated occurring between Aug 31 and September 14, 2010, which accounted for 80.6% (25/31) of all dengue cases (24 laboratory confirmed and 7 clinically diagnosed cases, and 31 in total)

The first case was traced and confirmed by laboratory diagnosis for positive IgM and IgG antibodies against dengue virus, with the symptoms of fever, headache, pharyngalgia and myalgia. The index patient returned to Dongguan on July 13, 2010 from a trip to Singapore and Malaysia, the symptoms appeared on July 22, 2010. After being treated as a cold case, the patient was recovered and left the hospital on July 26, 2010. Other 30 cases gradually surfaced in the same community from August 23, 2010 to September 14, 2010. Apparently, this dengue endemic in Dongguan was a local outbreak likely triggered by an imported case from Southeast Asia. The dengue cases concentrated emerging between Aug 31 and Sep 14, which accounted for 80.6% (25/31) of all dengue cases (Figure 1).

### Clinic/laboratory diagnosis and manifestations

The reported symptoms and signs in the 31 cases were fever (87.0%), headache (96.8%), rash (32.2%), myalgia (22.6%), hemorrhage (bleeding spots under the skin) (55.0%), arthralgia (6.5%), fever + headache + rash + myalgia (9.68%). In 24 of the laboratory confirmed cases, 12 of which were confirmed with real-time PCR; the other 12 were confirmed with IgG and IgM antibody detection in the serum; 58.3% of the laboratory diagnosed cases showed leucopenia, and 70.8% presented with thrombocytopenia (Table 2). Male patients comprised 58% of the cases. The youngest patient was 2 years old and the oldest was 82 years old, the median age was 28. About 29% of all patients were in the young age group (20~29 year old). All the cases were confined to Xintang community in Dongguan city.

**Table 2:** Symptom composition of 31 Cases of dengue fever patients in Xintang community, Dongguan city

Symptoms	Number of cases	Proportion (%)	Diagnosis methods
Fever	27	27/31 (87.1)	Clinically and laboratory diagnosed
	
Headache	30	30/31 (96.8)	
	
Rash	10	10/31 (32.2)	
	
Myalgia	7	7/31 (22.6)	
	
Hemorrhage (bleeding spots under the skin)	5	5/31 (16.1)	
	
Arthralgia	2	2/31 (6.5)	
	
Fever + Headache + Rash + Myalgia	3	3/31 (9.68)	

leucopenia	14	14/24 (58.3)	Laboratory diagnosed
	
thrombocytopenia	17	17/24 (70.8)	

### Virus identification

Three dengue virus isolates were successfully isolated from different patients' serum after suckling mice intracerebral inoculation and then culturing with C6/36 cells, which were named as DG2010, D10029-DG and D10030-DG. DG 2010 was isolated in Southern Medical University, the other two were isolated in Guangdong province CDC. These isolates were sequenced at E-protein region. The sequences of DG2010 and D10029-DG were totally same as D10030-DG, so were not submitted to GenBank. D10030-DG E-protein cDNA (GenBank Accession No: JN029811, onset date of the patient is Sep 6, 2010) was used for phylogenetic analysis.

**Figure 2:**
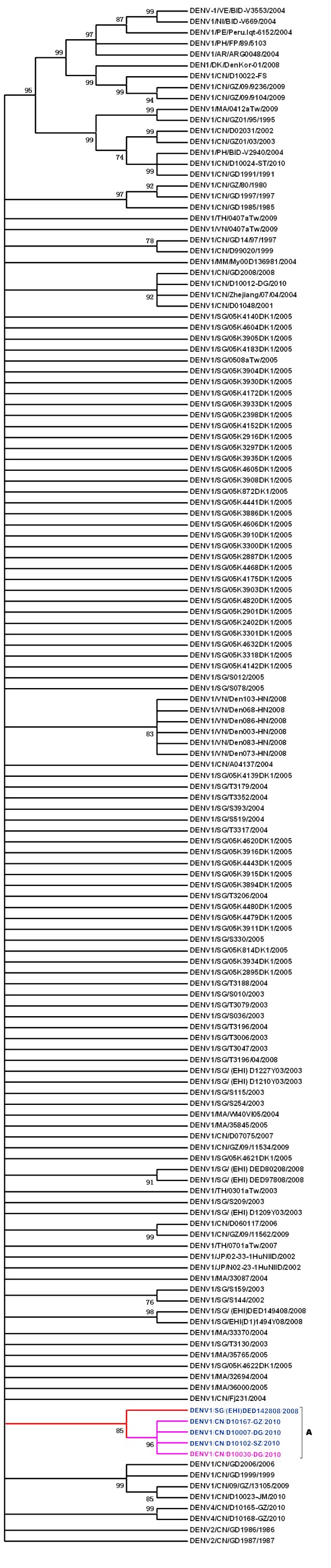
**Molecular Phylogenetic analysis of dengue virus isolates from this study compared with the other established dengue virus based on the alignment of E-protein gene**. Phylogenetic analysis were conducted in MEGA5. The evolutionary history was inferred by using the Maximum Likelihood method based on the General Time Reversible model. The bootstrap consensus tree inferred from 1000 replicates is taken to represent the evolutionary history of the dengue virus isolates analyzed. Branches corresponding to partitions reproduced in less than 50% bootstrap replicates are collapsed. The percentage of replicate trees in which the associated dengue virus isolates clustered together in the bootstrap test (1000 replicates) are shown next to the branches. Initial tree(s) for the heuristic search were obtained automatically as follows. When the number of common sites was < 100 or less than one fourth of the total number of sites, the maximum parsimony method was used; otherwise BIONJ method with MCL distance matrix was used. The analysis involved 141 nucleotide sequences. There were a total of 1427 positions in the final dataset. The cDNA of DG2010, D10029-DG and D10030-DG in E-protein region shared 100% identity with each other. The molecular phylogeny analysis result indicated that the D10030-DG isolate has a nearest relationship with DENV1/D10167-GZ/2010 (GenBank: JN029814.1), DENV1/D10102-SZ/2010 (GenBank: JN029813.1), and DENV1/D10007-DG/2010(GenBank: JN029807) (Figure 2), which were isolated in Guangdong in 2010, and didn't cause local outbreak. This cluster is statistically supported (bootstrap value = 96%) and is within a clade contain the sequence DENV1/SG/(EHI)DED142808/2008, isolated in Singapore in 2008 also statistically supported (bootstrap value = 85%)

Phylogenetic analysis showed the D10030-DG isolate closely related to DENV1/D10167-GZ/2010 in a cluster contains DENV1/D101102-SZ2010, DENV1/D10007-DG/2010 and DENV1D1067-GZ/2010 also, (Figure 2) which were isolated in Guangdong in 2010, and didn't cause local outbreak. This cluster is statistically supported (bootstrap value = 96%) and is within a clade contain the sequence DENV1/SG/(EHI)DED142808/2008, isolated in Singapore in 2008 also statistically supported (bootstrap value = 85%).

### Enhanced fever surveillance after outbreak reporting

**Figure 3:**
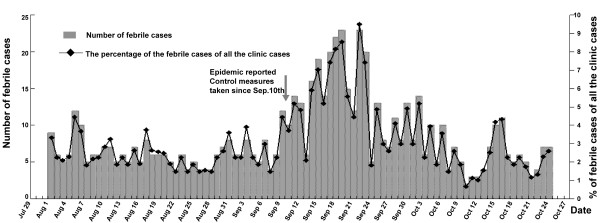
**Surveillance of febrile cases in Qifeng clinic during the dengue outbreak**. According to the report form Qifeng clinic near Xintang community, the number of the febrile cases peaked between September 12 and September 23, 2010. The febrile cases decreased apparently after September 23, 2010

Subsequent to the outbreak, an extra observation site was set up in Qifeng clinic near Xintang community. According to the data set from Dongguan CDC, the number of the febrile cases peaked between September 12 and September 23, 2010, and decreased apparently after September 23 (Figure 3). Not all of the febrile cases but only those suspected dengue cases were tested for dengue with laboratory techniques. Since September 14, 2010, there were no more suspected cases reported. The peak time for the febrile cases detected in the fever surveillance was late than the peak time for dengue cases reported (Figure 1 and Figure 3).

### Vector surveillance and control measures

**Figure 4:**
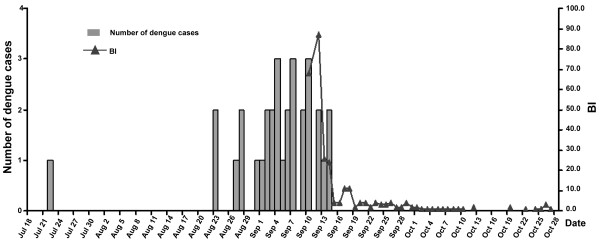
**The temporal dynamics of dengue cases and Aedes albopictus prevalence during the dengue outbreak in Dongguan**. Since September 10, 2010, measures have been taken to control the mosquitoes, such as cleaning out ponds to minimize breeding places, and spraying insecticides to kill the mosquitoes. The Breteau Index (BI) dropped greatly from 68 on September 10, to 4 on September 15, and 2 on September 19. The BI has been stably maintained at 5 or less since September 19. The dengue case was not reported any more since September 14, 2010

Since September 10, 2010, measures have been taken to control the *Ae. albopictus*, such as cleaning out ponds to minimize breeding places, and spraying insecticides to kill the mosquitoes. From September 10 to October 26, 2010, about 5000 families cooperated to mosquito control activities, and about 1000 families participated in indoor sterilization. Specifically, about 10,000 water containers were cleaned out; 120,000 m^2 ^outdoor areas were treated with insecticides; about 10,000 m^2 ^outdoor areas (such as grassy areas and ponds) were cleaned out. The Breteau Index (BI) dropped from 68 on September 10, to 4 on September 15, and 2 on September 19. The BI has been stably maintained at 5 or less since September 18. There was no more dengue cases reported since September 14, 2010 (Figure 4). As the every-day vector surveillance started only from Sep. 10, 2010, the BI at the beginning of the outbreak was unknown.

## Discussion

This dengue fever epidemic in Dongguan occurred within a restrained time and place, had a concentrated distribution of cases. According to the analysis of clinical manifestations, laboratory detection results, epidemiology characteristics, and *Ae. albopictus *breeding dynamics, this endemic dengue was caused by an imported case, transmitted by *Ae. albopictus *and resulted in a local outbreak. The source of infection may be from Malaysia based on the genotyping analysis and the travel history of the index case.

In Dongguan city, *Ae.albopictus *is the only vector for dengue. The difference of vectorial capacity for human dengue transmission between *Ae. albopictus *and *Ae. Aegypti *is still controversial. *Ae. aegypti *mostly only feed on humans, but *Ae. albopictus *take blood from a variety of animal species even though both species feed readily on humans. The amplification rate of DENV in *Ae. albopictus *is lower than in *Ae. aegypti*. These characteristics decrease *Ae. albopictus*' efficiency as a dengue vector [6]. On the contrary, some researchers agreed that infection and dissemination rates were not significantly different between *Ae. albopictus *and *Ae. aegypti *when exposed to high titers of DENV-2 virus (10^8.1 ^mosquito infectious dose 50/mL and 10^7.5 ^plaque forming units/ml). When *Ae. albopictus *outnumbered *Ae. aegypti, Ae. albopictus *acted as the major vector of dengue virus [16].

During this outbreak of dengue, there was also a limitation in the fever surveillance, not all of the febrile cases but only those dengue suspected cases were sent for laboratory confirmation. Dengue virus infection can cause a wide range of clinical manifestations, and many infections do not cause any or only very mild symptoms. About 2 weeks after the peak time of dengue cases reported, a peak time for febrile cases occurred (Figure 1 and Figure 3). It was not guaranteed that those febrile cases were not dengue virus infected. The reported 31 dengue cases represent only the peak of the iceberg of dengue virus infections, but do not include those mild symptom cases or those asymptomatic infections without going for health care consultations. A follow-up sera survey might be of interest to estimate the true attack rate in the community.

Several reasons for this outbreak of dengue in Dongguan should be alerted. First, it was triggered by an imported case which was misdiagnosed. Dengue is not a major infectious disease in China. Some of the doctors in Xintang community, where this dengue outbreak occurred, were from North of China where there is a lack of experience in dengue diagnosis and therapy. Dengue case had unspecific clinical manifestations, presenting with symptoms common to cold, typhoid fever, or paratyphoid fever [17]. The misdiagnosis of the dengue case at the early endemic stage might have contributed to a higher chance of the subsequent outbreak. Second, the residents' life habits and environment were suitable for the breeding of *Aedes albopictus*. Specifically, the residents in this community have a habit of culturing water plants and keeping water for watering flowers, and fail in mosquito prevention and extermination. Finally, a lack of awareness for self-prevention against dengue greatly contributed to its transmission.

The impact of meteorological factors on the epidemic of vector-borne diseases has recently received more attention [4,18]. The seasons for dengue outbreak are concentrated in summer and autumn, which is a peak time for *Aedes *breeding due to the high temperature and sufficient rainfall. In line with this point, this dengue outbreak in Dongguan occurred in summer, the density of *Ae. albopictus *mosquitoes were highly above the threshold. Normally, a high risk of dengue outbreak is associated with a BI that exceeds 20 [3]. The BI on Sep 10, 2010 in Xintang community had reached 68.

Dongguan is characterized by its fast urbanization in south China. Several characterized points of urbanization contributed to this outbreak of dengue in Dongguan: the residents are highly concentrated; the residents' life habits help to form the habitats of *Ae. albopictus*; the high density of *Ae. albopictus*. An imported dengue case successfully brought the outbreak in this area under this suitable situation [19].

Based on the present situation in China, dengue control should be improved and focused on these aspects: 1) Strengthening the training of medical personnel for dengue rapid diagnosis and therapy at both school level and health system level; 2) Enhancing the control measures or system to maintain the *Aedes *breeding under a safe density level (BI < 5), [3] such as more frequent BI investigation. 3) Increasing the awareness of dengue epidemics in other countries and establishing health instructions for the international travelers; 4) Improving the conventional fever surveillance, diagnosis of febrile cases, and the timely report of the disease once a causative pathogen is identified; 5) Increasing the prompt reaction to control the diseases spreading, such as controlling vector density, isolating the infected case and applying effective self-prevention methods.

## Conclusions

Since the first outbreak of dengue was documented in Guangdong province in 1978 in China, the epidemic of dengue were reported sequentially in Southern China. From Jul.22, 2010 to September 14, 2010, a dengue triggered by an imported case broke out in Xintang community in Dongguan, China and was transmitted by *Ae. albopictus*. 31 cases were confirmed and reported. The time span of outbreak was 55 days, and the infection rate was 50.63 out of a population of 100 000. Dongguan city has been undergoing its fast urbanization and the consequent factors contributed to this dengue outbreak: the highly concentrated population; the high density of *Aedes albopictus *mosquitoes; unplanned constructed houses lack of mosquito prevention facilities. The misdiagnosis of the first imported case might improve the chance of the outbreak of dengue. The timely reaction to this dengue outbreak had restrained its epidemic in Xintang community. This report has reaffirmed the importance of a surveillance system for infectious diseases control and aroused the awareness of an imported case causing the epidemic of an infectious disease.

## Competing interests

The authors declare that they have no competing interests.

## Authors' contributions

HJP paper writing, data analysis, sequence alignment. HBL site investigation, data collection and organization. QLZ site investigation and clinic cases information collection. BYX site investigation, data collection and organization. HZ laboratory investigation. WHL vector investigation. WZ clinical data and sample collection. YPZ instruction of site investigation and data analysis. XGZ site investigation, clinic cases information collection and vector surveillance. SJ laborotary diagnosis and sequencing. JHD vector investigation. GYY data analysis. JFH site investigation, data collection and organization and paper writing. XGC site investigation, data collection and organization and paper writing.

## Pre-publication history

The pre-publication history for this paper can be accessed here:

http://www.biomedcentral.com/1471-2458/12/83/prepub
